# The Landscape of Women's Health Podcasts: A Scoping Review

**DOI:** 10.1111/jog.70483

**Published:** 2026-09-01

**Authors:** Ellen Beatriz Saboia de Castro, Janielle Ferreira de Brito Lima, Kayo Elmano Costa da Ponte Galvão, Eremita Val Rafael, William Caracas Moreira, Roger Rodrigues da Silva, Mônica Oliveira Batista Oriá, Maria Luziene de Sousa Gomes

**Affiliations:** ^1^ Department of Nursing Universidade Federal do Maranhão São Luís Maranhão Brazil; ^2^ Department of Nursing Universidade Federal do Ceará Fortaleza Ceará Brazil

**Keywords:** digital media, information technology, medical education, podcasts, women's health

## Abstract

**Aim:**

To map and characterize the available evidence on podcasts addressing topics related to women's health.

**Methods:**

This scoping review was conducted in accordance with the Joanna Briggs Institute (JBI) guidelines. A comprehensive search was carried out in 12 databases in November 2025. Quantitative and qualitative studies addressing podcasts related to women's health were included, with no restrictions on publication date or language. The review protocol was registered on the Open Science Framework (https://osf.io/7p9yr/overview). The results are reported in accordance with the PRISMA extension for Scoping Reviews (PRISMA‐ScR).

**Results:**

A total of 37 studies were included, primarily targeting health professionals and students (*n* = 15). Thematic analysis showed that gynecology was the most frequent category (48.6%), with subthemes focusing on the climacteric (*n* = 6), breast cancer (*n* = 4), and fertility (*n* = 3). Breastfeeding and healthy lifestyle shared second place (18.9% each), emphasizing lactation challenges and physical activity/nutrition, respectively. Obstetrics (13.5%) addressed labor and postpartum recovery, while motherhood (8.1%) explored maternal guilt. Finally, miscellaneous themes (16.2%) covered mental health, chronic diseases, and maternal mortality.

**Conclusion:**

Podcasts have emerged as a versatile and strategic tool for health education and self‐care promotion throughout the female life cycle. While widely used for professional training and patient empowerment, there is a critical need for rigorous content curation and evidence‐based production to mitigate the risks of misinformation. Future research should focus on validating these digital tools and evaluating their long‐term impact on clinical outcomes and health literacy.

## 1 Introduction

The use of podcasts has grown remarkably in recent years [1]. According to recent data, there are 504.9 million podcast listeners worldwide, representing 23.5% of all internet users [2]. A podcast is a digital audio or video file created and made available on online platforms, allowing sharing and access through mobile devices such as smartphones, providing flexibility and autonomy in obtaining information. It is, therefore, a modern form of communication that combines accessibility, portability, and practicality [1, 3]. The most widely used platforms globally are Spotify and Apple Podcasts [2].

Podcast audiences display diverse profiles in terms of both demographics and interests. Sweden leads the global ranking of podcast consumption (47%), followed by Brazil and the Republic of Ireland, both exceeding 40% listenership [2]. One of the main advantages of this format is convenience, as episodes can be listened to at any time or place, even during daily activities [4]. Furthermore, podcast creators come from a wide range of backgrounds, from healthcare professionals to laypersons interested in communication and health education [3]. Thus, podcasts have emerged as a dynamic, engaging, and accessible medium for communicating health content and supporting the learning process of diverse audiences [5].

In the educational field, particularly in health training contexts, podcasts have stood out as a complementary tool in the teaching‐learning process. In an increasingly digital and globalized world, they have become valuable allies for medical and multiprofessional education, offering open, free, and flexible access to knowledge. Several studies indicate that podcasts can support students, reinforce theoretical content, foster active learning, and promote professional development [6, 7, 8]. Due to their simplicity and wide dissemination, podcasts have proven to be an effective pedagogical strategy, facilitating communication and disseminating scientific information in an accessible and interactive way [3].

Beyond their educational role, podcasts have established themselves as a significant medium for scientific and health communication, with the potential to influence listeners' health behaviors and outcomes [4]. This innovative digital health promotion tool enables the reach of broad and diverse audiences, enhancing engagement, understanding, and knowledge retention, particularly when combined with factors such as content format, expert participation, and interactivity [9]. Thus, podcasts make a substantial contribution to the dissemination and accessibility of health information, fostering knowledge and individual empowerment in self‐care management by simplifying the educational and communicative process in health [10].

In this context, podcasts have played an increasingly important role in health education and promotion. Women's health, in particular, stands out as a topic of great global importance, encompassing aspects ranging from reproductive and maternal health to chronic diseases and mental well‐being. A preliminary search for existing reviews on the use of podcasts in the field of women's health was conducted, and no reviews were identified. Therefore, this scoping review aims to map and characterize the available evidence on podcasts addressing topics related to women's health.

## 2 Methods

We followed the Joanna Briggs Institute (JBI) guidelines for conducting this scoping review [11]. The following steps will be used: (1) define and align the aim and research question; (2) develop and align inclusion criteria with the aim and question; (3) describe the planned approach to evidence search, selection, data removal, and presentation of evidence; (4) search for evidence; (5) select evidence; (6) extract evidence; (7) analyze evidence; (8) present results; and (9) summarize the evidence in relation to the purpose of the review, draw conclusions, and note any implications of the findings [11].

The details for these methods in a predefined protocol at the Open Science Framework [12] (available at https://osf.io/7p9yr/overview). We reported this review following the PRISMA‐Scr extension for scoping reviews [13] (Supporting Information Data S1).

### 2.1 Research Question

Specifically, this scoping review aims to answer the following question: What evidence is available in the scientific literature on podcasts related to women's health targeting both the female population and healthcare providers?

The research question was formulated using the PCC framework (Population, Concept, Context). In this approach, P (Population) refers to women in different stages of life and health contexts and/or health professionals and students involved in women's healthcare; C (Concept) concerns podcasts as a means of communication, education (professional or patient‐centered), and health promotion; and C (Context) encompasses women's health care at different levels of attention, with no geographical restriction (Supporting Information Data S2).

### 2.2 Eligibility Criteria

Eligible sources included articles published that address podcasts related to women's health, based on population data from any geographical region. Primary studies with quantitative, qualitative, or mixed‐methods approaches will be considered, with no restrictions on publication period or language. Opinion papers, experience reports, ongoing clinical trials, incomplete articles, book chapters, editorials, protocols, and procedures were excluded.

### 2.3 Search Strategy

#### 2.3.1 Electronic Searches

Searched the following databases from the inception of each resource to the date of the search (27 November 2025): MEDLINE/PubMed (via the National Library of Medicine), Web of Science, Scopus, CINAHL (EBSCO), Cochrane Library, EMBASE (Elsevier), and the Latin American and Caribbean Literature in Health Sciences (LILACS) via the Virtual Health Library (VHL). All databases were accessed through the CAPES Journal Portal (Coordination for the Improvement of Higher Education Personnel).

Free‐text terms were searched in the title, abstract, and keyword fields. Within each block, synonyms and spelling variants were combined with the Boolean operator “OR,” and the two blocks were combined with “AND.”

Prior to the main search, a preliminary exploratory search was conducted in MEDLINE (via PubMed) to identify the terminology most frequently used to describe podcasts and women's health in the relevant literature. This preliminary step confirmed that the terms “Podcast,” “Podcasts,” and “Webcast” were the most consistently applied in indexed titles, abstracts, and author keywords; these were therefore adopted as the core free‐text terms for the concept block, combined with population terms through the Boolean operators.

Controlled vocabulary was not applied uniformly across databases: Emtree subject headings were used only in Embase, whereas the remaining databases relied on free‐text terms without controlled vocabulary (e.g., MeSH or CINAHL subject headings). A preliminary strategy was first developed in MEDLINE (via PubMed) and subsequently adapted to the syntax of each database and gray‐literature source. The search was checked against the PRESS checklist [14].

The complete search string executed in every database and source, together with the date of the search and the number of records retrieved, is reported in the Supporting Information Data S3 and S4.

#### 2.3.2 Complementary Searches

In addition, complementary searches were conducted in Google Scholar, the Brazilian Digital Library of Theses and Dissertations (BDTD), the Catalog of Theses and Dissertations (CTD) of the Coordination for the Improvement of Higher Education Personnel (CAPES), Open Access Theses and Dissertations (OATD), and ProQuest to gather additional data on studies not published or indexed in electronic databases.

All information sources, databases, and complementary searches were accessed on the same day to minimize potential selection bias. Initially, a preliminary search was conducted in the MEDLINE database via PubMed, and the search strategy was subsequently adapted for use in the other databases and complementary sources. Finally, the literature search was completed by screening the reference lists of all included studies to identify additional relevant publications. The complete search strategy is available in the Supporting Information Data S3 and S4.

### 2.4 Data Collection and Extraction

Search results from each database were imported into the Rayyan reference management platform [15]. Two reviewers (EBSC and MLSG) independently screened titles and abstracts to identify potentially relevant studies. In the subsequent stage, the full texts of the preselected articles were assessed to confirm eligibility. For complementary sources, the screening and selection process was conducted manually. Titles, abstracts, and full texts identified through complementary searches were independently screened by the same two reviewers, following the same eligibility criteria and review procedures applied to the database searches. Any disagreements were resolved through consensus or, when necessary, by consultation with a third review author (MOBO). The reasons for excluding studies that were considered potentially eligible were documented in the PRISMA flow diagram.

A standardized data extraction form was developed and pilot‐tested in advance. For studies that met the inclusion criteria, two review authors (EBSC and MLSG) independently extracted the following information: author, year of publication, country; aim(s) of study; method (type of study, sample, and production); target audience and main results (Supporting Information Data S5).

### 2.5 Analysis and Presentation of the Data

The results were analyzed using a combination of quantitative descriptive analysis and qualitative thematic content analysis. Quantitative data were summarized using descriptive statistics, including absolute and relative frequencies, to characterize the volume and types of podcasts related to women's health. Qualitative data were analyzed using thematic content analysis, a systematic method for identifying, organizing, and interpreting recurring patterns or “meaning units” within the extracted data. This approach enabled the identification and mapping of key themes aligned with the objectives of this scoping review.

The findings were presented through narrative summaries, detailed tables, and figures. In accordance with the methodological framework of a scoping review, no formal critical appraisal or risk‐of‐bias assessment of the included studies was conducted. Finally, the analysis identified and discussed the most frequently addressed topics as well as notable gaps in the literature, providing directions and recommendations for future research.

## 3 Results

**FIGURE 1:**
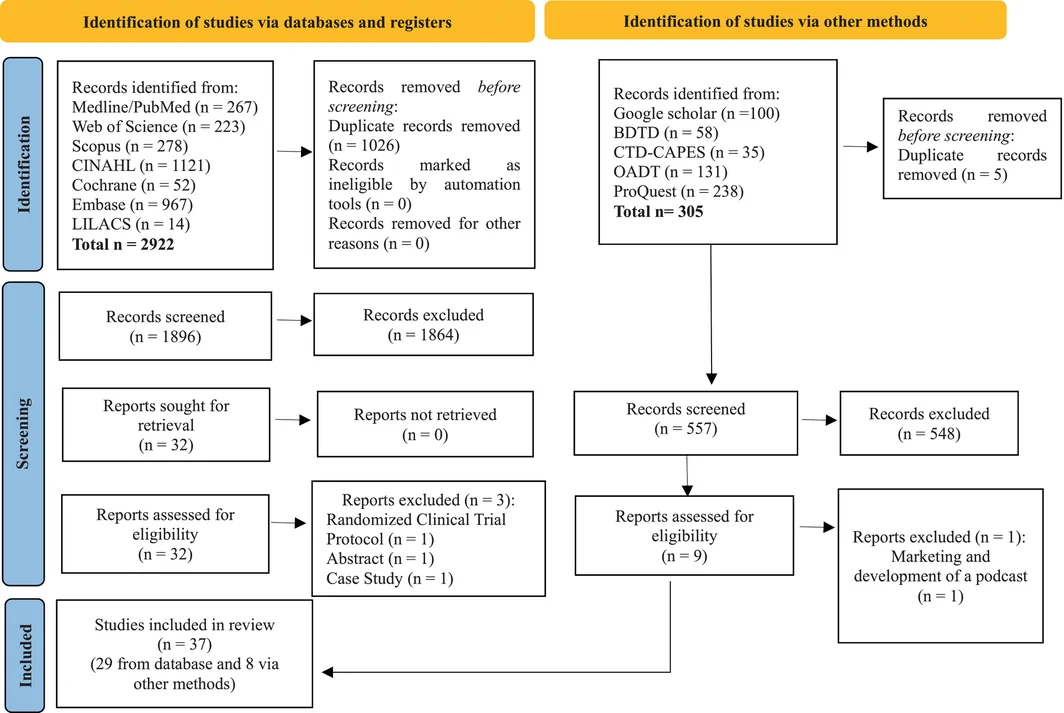
PRISMA flow diagram.

The database search identified 3484 records. Of these, 1031 duplicate records were removed, resulting in 2453 unique records. Titles and abstracts of these 2453 records were screened according to the eligibility criteria, and 2412 records were excluded. A total of 41 studies were selected for full‐text review, of which 37 were included in this scoping review: 29 studies identified through database searches and 8 identified through other methods. Figure 1 illustrates the selection process using the PRISMA diagram.

### 3.1 Study Characteristics

Regarding the time frame, the publications spanned the period from 2009 to 2025. Among the 37 included studies, most were published in 2023 (*n* = 8). With respect to country of origin, the studies were conducted predominantly in the United States of America (*n* = 18). In terms of methodological approach, the studies were mainly characterized as methodological and qualitative (*n* = 8). The predominant target audience consisted of health professionals and students (*n* = 15). Table 1 summarizes the main characteristics of the included studies.

**TABLE 1:** Characteristics of included studies (*n* = 37).

Characteristics	*n*	%
*Year of publication*		
2025	7	18.9
2024	6	16.2
2023	8	21.6
2022	7	18.9
2021	4	10.8
2020	1	2.7
2018	2	5.4
2015	1	2.7
2009	1	2.7
*Country of study*		
Australia	1	2.7
Brazil	9	24.3
Canada	1	2.7
Denmark	1	2.7
United States of America	18	48.6
Egypt	1	2.7
Iran	2	5.4
Ireland	2	5.4
Nigeria	1	2.7
United Kingdom	1	2.7
*Type of study*		
Evaluative	1	2.7
Experimental	6	16.2
Methodological	8	21.6
Mixed methods	3	8.1
Qualitative	8	21.6
Quase‐experimental	5	13.5
Metanarrative review	1	2.7
Cross‐sectional	5	13.5
*Target audience* ^a^		
Women	6	16.2
Teenagers/young people	3	8.1
Pregnant women	4	10.8
Hypertensive people	1	2.7
Mothers aged 25–55	1	2.7
Women with visual impairments	1	2.7
Women with epilepsy	1	2.7
Overweight women	3	8.1
Women after mastectomy	1	2.7
Women in menopause, perimenopause, and postmenopause	4	10.8
Women in the perinatal period	1	2.7
Postpartum women	3	8.1
People with uterine fibroids	1	2.7
Professionals and students in the health field	15	40.5
General public	3	8.1
Residents in gynecology and obstetrics	2	5.4

^a^ The total number exceeds the number of studies included (*n* = 37), since some studies involved data from more than one target audience.

### 3.2 Podcast Themes

The thematic analysis revealed a broad diversity of topics concerning women's health. Gynecology emerged as the predominant category, accounting for 48.6% of the records, with a primary focus on the climacteric (perimenopause/menopause), breast cancer, and fertility issues. Tied for second place at 18.9% each were the breastfeeding and healthy lifestyle categories, which addressed lactation challenges and physical habits, respectively. Obstetrics represented 13.5% of the sample, while motherhood (8.1%) and miscellaneous themes (16.2%) covered psychosocial aspects and broader health issues, such as maternal mortality and mental health.

The following table (Table 2) provides a comprehensive breakdown of these themes and subthemes, supported by the included literature.

**TABLE 2:** Thematic categories and subthemes of podcasts related to women's health (*n* = 37).

Themes and subthemes	References
*Gynecology*	
Perimenopause/menopause	[16, 17, 18, 19, 20, 21]
Breast cancer	[17, 22, 23, 24]
Sexual health/Sexual education	[17, 24, 25]
Fertility	[21, 26, 27]
Cervical cancer	[17, 21]
Endometriosis	[17, 28]
Vaginal infections	[29, 30]
Assisted reproduction with egg donation	[26]
Gynecological disorders	[21, 30]
Breast self‐examination	[31]
Urogenital and breast disorders	[21]
Contraception	[21]
Hormone replacement therapy	[17]
Stress urinary incontinence (SUI)	[32]
Pelvic floor muscle training (PFMT)	[32]
Surgery for SUI	[32]
Diagnosis and treatment of uterine fibroids (UF)	[33]
Diagnosis and treatment of polycystic ovary syndrome (PCOS)	[27]
Menstrual disorders	[27]
Reconstructive surgery	[21]
*Obstetrics*	
Complications of labor and delivery	[21, 34]
Postpartum recovery	[21, 34]
Weight stigma in prenatal care	[35]
Prenatal care	[21]
First‐trimester pregnancy loss	[21]
Anesthesia for labor	[34]
Labor induction	[34]
Vaginal delivery	[34]
Reasons for performing a cesarean section	[34]
Second stage of labor	[34]
Newborn care	[21]
Maternal‐fetal medicine	[21]
Gestational loss	[36]
Embryonic human development	[37]
Fetal development	[37]
Physiological and anatomical changes	[37]
*Breastfeeding*	
Importance of breastfeeding	[21, 38, 39, 40, 41]
Breastfeeding challenges	[38, 39, 40, 42, 43]
Benefits of breastfeeding	[21, 39, 40]
Breastfeeding techniques	[38, 39, 40]
Breast problems	[38, 39]
Breastfeeding consultancy	[40, 43]
Importance of the support network	[38, 39]
Milk expression and storage	[38]
Myths and truths	[38]
Maternal nutrition and rest	[39]
Human Milk Banks in Brazil	[40]
*Healthy lifestyle*	
Physical activity	[16, 17, 44, 45, 46, 47]
Healthy eating	[17, 44, 45, 46, 47]
Healthy gestational weight gain	[45, 46, 47]
Weight loss	[47, 48]
*Motherhood*	
Maternal guilt	[40, 42]
Challenges of motherhood	[42]
Puerperium	[42]
Motherhood and work	[42]
Maternal fatigue and exhaustion	[42]
Motherhood during quarantine/pandemic	[42]
Dismissal during pregnancy	[42]
Gestation	[41]
Baby sleep routines	[41]
Newborn Care (oral hygiene, vaccination, and pacifier)	[41]
Formula feeding	[41]
Parenting	[41]
*Other topics*	
Mental health	[46, 49]
Epilepsy from the preconception period to the postpartum period	[50]
Self‐care in systemic arterial hypertension	[51]
Geriatric care	[21]
Maternal mortality rates	[52]
Collaborative care to improve women's health	[52]

Abbreviations: PCOS: polycystic ovary syndrome; PFMT, pelvic floor muscle training; SUI, stress urinary incontinence; UF, uterine fibroids.

**FIGURE 2:**
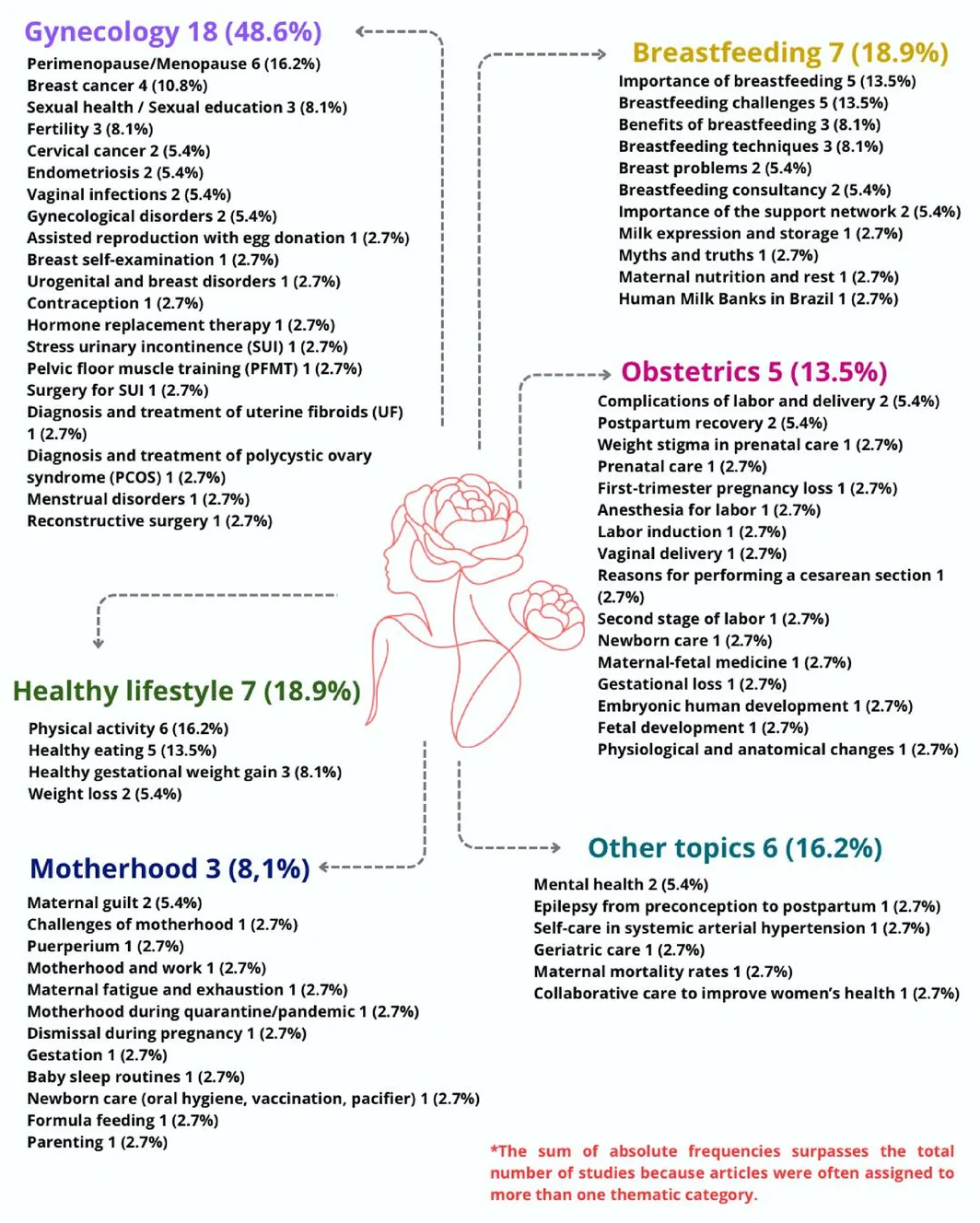
Visual synthesis of the thematic distribution and relative frequency of podcast categories in women's health.

While the table offers a detailed textual map of each subtheme and its corresponding references, Figure 2 provides a comprehensive visual synthesis of these findings. It illustrates the prevalence of each thematic category and their relative frequencies, facilitating the identification of critical trends and gaps within the women's health podcast landscape.

## 4 Discussion

This scoping review aimed to map the existing literature on podcasts within the context of women's health, providing a comprehensive overview of explored themes, target audiences, and directions for future research. The findings reveal a predominant concentration in the field of gynecology, followed by breastfeeding and healthy lifestyle. Notably, a key finding of this study is the scarcity of research evaluating the authorship and clinical quality of this content, highlighting a critical gap to be addressed.

The thematic diversity identified reflects the growing and multifaceted use of podcasts, which currently range from classic clinical topics to psychosocial and educational dimensions [31, 44]. Recent studies highlight the potential of these digital audio resources in promoting self‐care and behavioral change, particularly among adult women [16]. In this context, platforms like Spotify emerge as innovative approaches to digital health education, fostering women's independence in managing their own care [53].

The impact of these tools is further enhanced by narrative formats, which have proven effective in demystifying topics such as reproductive health, menopause, endometriosis, and sexual rights. By immersing the listener in real stories, podcasts facilitate the processing of complex information and help overcome behavioral barriers [27, 28, 54]. This narrative approach fosters deeper engagement, strengthening the intention to adopt healthy habits [55].

A clear example of this engagement is seen in the approach to menopause and perimenopause. The concentration of content on this theme reflects an attempt to fill a historical gap in health education, often marked by stigmas and communication failures in clinical settings [56]. Podcasts facilitate the validation of experiences and reduce the isolation felt by women during this stage, allowing continuous access to information outside the clinical environment [17]. Similarly, in clinical gynecology, this media excels by integrating technical information with patient reports on conditions like endometriosis [28] and uterine fibroids [33], though the variation in information quality reinforces the need for evidence‐based curation [57].

Digital support also extends to the pregnancy‐puerperal cycle. In Obstetrics, podcasts aid in prenatal education and childbirth preparation, increasing perceived control and autonomy for pregnant women [45]. Breastfeeding also stands out, with evidence suggesting that audio interventions increase maternal self‐efficacy and confidence, particularly when tailored to specific audiences, such as first‐time mothers or women with visual impairments [38, 39].

Beyond specific clinical issues, the audio format benefits the promotion of healthy lifestyles due to its flexibility. Integrating information consumption into daily routines encourages adherence to physical activity and nutrition guidelines across different life stages [16, 46]. This behavior of seeking health information outside traditional services is a global trend, with women utilizing social media and podcasts to address concerns [58].

However, this increase in digital consumption brings significant challenges. While digital media democratizes knowledge, it can also facilitate the spread of incorrect information, posing risks to public health [59]. In this context, health literacy becomes a core competency, as it is essential for users to critically filter and apply information [60].

Regarding the target audience, the results reveal that podcasts have consolidated as a strategic mobile learning tool for health professionals and students. The use of this media in undergraduate and continuing education promotes access to updated content and active methodologies, enhancing clinical decision‐making and professional confidence [5, 10]. The feasibility of this approach is demonstrated by resident‐led initiatives, which show that podcasts created by peers can effectively support medical education in specialized fields like Obstetrics and Gynecology [21]. Furthermore, integrating such digital tools into multispecialty curricula has proven essential to optimize training in areas such as menopause management, thereby enhancing practitioners' knowledge and their preparedness to provide patient‐focused care [18].

Ultimately, the rise of podcasts in women's health aligns with the WHO “Global Strategy on Digital Health 2020‐2025.” By promoting equity, sustainability, and patient empowerment through accessible technologies, podcasts become allies in the pursuit of universal health coverage [61]. Nevertheless, to reach their full potential, it is imperative to strengthen regulatory frameworks and data security, ensuring this digital innovation remains ethical and evidence‐based.

A major strength of this study is the adoption of a rigorous methodological path based on the JBI guidelines and the PRISMA‐ScR checklist. Furthermore, the search strategy was robust, covering multiple international databases and gray literature without language or time restrictions. This approach allowed for an exhaustive mapping of the scientific landscape of women's health podcasts, identifying trends, target audiences, gaps, and emerging areas of research.

Despite these strengths, some limitations must be considered. The search relied predominantly on free‐text terms and did not apply controlled vocabulary (e.g., MeSH or CINAHL subject headings) consistently across databases. This, together with the specific term set adopted, may have limited retrieval; relevant records indexed only under subject headings such as “Webcasts” may not have been captured. Significant heterogeneity was observed among the included studies, with variations in methodological designs, target audiences, and production formats. This diversity makes direct comparison difficult and prevents deeper analytical synthesis. Additionally, as a scoping review, the focus was restricted to mapping and characterizing evidence; thus, clinical effectiveness, direct impacts, or specific health outcomes associated with podcast use were not evaluated.

## 5 Conclusion

This scoping review demonstrates that podcasts have established themselves as a strategic and versatile tool for disseminating information on women's health. The predominance of content focused on gynecology, with emphasis on the climacteric, breast cancer, and fertility, alongside the growth of themes such as breastfeeding and healthy lifestyles, reflects the utility of this media for both patient empowerment and the continuing education of healthcare professionals (*mobile learning*).

However, the rapid expansion of this digital technology occurs within a landscape of significant heterogeneity and a lack of systematic evaluations regarding the technical and scientific quality of the content produced. The concentration of evidence in developed countries and the absence of clear curation criteria highlight an urgent need for studies that evaluate the clinical efficacy of these resources and their impact on health literacy.

Ultimately, for podcasts to fulfill their role within the WHO Global Strategy on Digital Health, it is fundamental that healthcare professionals and regulatory bodies actively engage in the production and recommendation of evidence‐based content. Only then will it be possible to ensure that this tool for democratizing knowledge contributes, in a safe and ethical manner, to the promotion of women's health and autonomy.

## Author Contributions

**Janielle Ferreira de Brito Lima:** writing – original draft, methodology, writing – review and editing, formal analysis, data curation. **Mônica Oliveira Batista Oriá:** writing – original draft, methodology, writing – review and editing, software, formal analysis, data curation. **Eremita Val Rafael:** writing – original draft, methodology, writing – review and editing, formal analysis, data curation. **William Caracas Moreira:** writing – original draft, methodology, writing – review and editing, formal analysis, data curation. **Maria Luziene de Sousa Gomes:** conceptualization, investigation, writing – original draft, methodology, writing – review and editing, software, formal analysis, project administration, data curation, supervision. **Roger Rodrigues da Silva:** writing – original draft, methodology, writing – review and editing, formal analysis, data curation. **Kayo Elmano Costa da Ponte Galvão:** data curation, formal analysis, methodology, writing – review and editing, writing – original draft. **Ellen Beatriz Saboia de Castro:** conceptualization, investigation, writing – original draft, methodology, writing – review and editing, software, formal analysis, project administration, data curation.

## Funding

The authors have nothing to report.

## Ethics Statement

The authors have nothing to report.

## Consent

No written consent has been obtained from the patients as there is no patient identifiable data included.

## Conflicts of Interest

The authors declare no conflicts of interest.

## Supporting information


**Data S1:** PRISMA 2020 checklist.

**Data S2:** Eligibility criteria for the scoping review based on PCC.

**Data S3: and S4** Databases and complementary searches conducted.

**Data S5:** Extraction Form.

## Data Availability

The data that supports the findings of this study are available in the Supporting Information of this article.
