# Supplementary material for: The Landscape of Women's Health Podcasts: A Scoping Review
Source: J Obstet Gynaecol Res. 2026 Sep 1;52(9):e70483. doi: 10.1111/jog.70483 (PMC13533598; doi:10.1111/jog.70483)
Supplement: Supplementary file 1 [file JOG-52-0-s001.docx]

**Supplementary Material**

**Data S3:** Databases search conducted on November 27, 2025.

| **Database** | **Search strategy used** |
| --- | --- |
| MEDLINE/PubMed | (Women OR "Women's Health" OR "Womens Health") AND (Webcast OR Podcasts OR Podcast) |
| *Web of Science* |  |
| SCOPUS |  |
| CINAHL (EBSCO) |  |
| Cochrane |  |
| LILACS |  |
| EMBASE | ('women'/exp OR women OR 'womens health'/exp OR 'womens health') AND ('podcast'/exp OR podcast) |

**Data S4:** Complementary searches conducted on November 27, 2025.

| **Complementary searches** | **Search strategy used** |
| --- | --- |
| Google scholar | (Women OR "Women's Health" OR "Womens Health") AND (Webcast OR Podcasts OR Podcast) |
| ProQuest Dissertations and Theses |  |
| OADT |  |
| BDTD | Podcast AND Mulher |
| CTD-CAPES |  |

Abbreviations:  OADT, Open Access Theses and Dissertations; BDTD, Brazilian Digital Library of Theses and Dissertations; CTD, Catalog of Theses & Dissertations; CAPES, Coordination for the Improvement of Higher Education Personnel.
