# Supplementary material for: The Landscape of Women's Health Podcasts: A Scoping Review
Source: J Obstet Gynaecol Res. 2026 Sep 1;52(9):e70483. doi: 10.1111/jog.70483 (PMC13533598; doi:10.1111/jog.70483)
Supplement: Supplementary file 3 [file JOG-52-0-s003.docx]

**Supplementary Material**

**Data S2:** Eligibility criteria for the scoping review based on PCC (population, concept and context) approach.

| **Research question** | What evidence is available in the scientific literature on podcasts related to women's health targeting both the female population and healthcare providers? | | |
| --- | --- | --- | --- |
|  | **P** | **C** | **C** |
| **Extraction** | Women in different stages of life and health contexts and/or health professionals and students involved in women's healthcare | Podcasts as a tool for communication, education (professional or patient-centered), and health promotion. | Women’s health care at different levels of attention, with no geographical restriction |
| **Conversion** | Women  Women's Health  Womens Health | Webcast  Podcasts  Podcast | - |
| **Grouping** | Women; Women's Health; Womens Health | Webcast; Podcasts; Podcast | Identification through document reading |
| **Building** | Women OR "Women's Health" OR "Womens Health" | Webcast OR Podcasts OR Podcast | Identification through document reading |
| **Usage** | (Women OR "Women's Health" OR "Womens Health" AND Webcast OR Podcasts OR Podcast) | | |

Adapted from: Araújo WCO. Recuperação da informação em saúde: construção, modelos e estratégias. ConCI: Convergências em Ciência da Informação. 2020;3(2):100–134. doi: https://doi.org/10.33467/conci.v3i2.13447
