# Supplementary material for: The Landscape of Women's Health Podcasts: A Scoping Review
Source: J Obstet Gynaecol Res. 2026 Sep 1;52(9):e70483. doi: 10.1111/jog.70483 (PMC13533598; doi:10.1111/jog.70483)
Supplement: Supplementary file 4 [file JOG-52-0-s004.docx]

**Supplementary Material**

**Data S5:** Extraction Form

| **Author, year, and country** | **Aim** | **Method** | **Theme** | **Main results** |
| --- | --- | --- | --- | --- |
| Parnell et al., 2025  Ireland | To analyse the type and quality of online resources aimed at women with epilepsy, from pre-conception through the postpartum period. | Study Type: Metanarrative systematic review.  Sample: 4 podcasts Production: Not addressed.  Target Audience: Women with epilepsy from the preconception period through the postpartum period.  Location: Dublin, Ireland  Year: December 2023 to January 2024 | Epilepsy from the preconception period to the postpartum period. | The mean quality score for these 43 resources was 47.97 out of a maximum of 75, which is classified as “fair.”  Podcasts (n = 4) scored lower on five of the six study-specific questions: readability/audibility, language, sensitivity, accessibility, and value, compared with websites and videos.  Online resources for women with epilepsy are considered usable; however, they could be improved to enhance their overall quality. |
| Brito, 2025  Brazil | To develop an assistive technology, in the format of a podcast, on breastfeeding for people with visual impairment. | Study Type: Methodological study.  Sample: Women with visual impairment.  Production: The podcast developed was entitled *“Nurturing with All the Senses”*, consisting of 12 episodes with a total duration of 72 minutes and 20 seconds. The podcast was recorded in a professional studio, ensuring high-quality audio production and language appropriate for the target audience.  Target Audience: Women with visual impairment and healthcare professionals.  Location: Redenção and Fortaleza, Ceará, Brazil.  Year: September 2023 to March 2025. | Breastfeeding  Importance of breastfeeding  Breastfeeding challenges  Breastfeeding techniques  Breast problems  Return to work (milk expression and storage)  Importance of the support network  Myths and truths about breastfeeding  Testimony of a mother with visual impairment who breastfed | The podcast stands out for its communicational, instrumental, methodological, and attitudinal accessibility, in addition to its positive impact on maternal and child health. By combining practical information on breastfeeding with testimonies from mothers with visual impairment, it is concluded that this is an accessible, engaging, and essential resource to support these women in their breastfeeding experience.  The podcast demonstrated potential to increase adherence to breastfeeding, prevent early weaning, and enhance the autonomy of women with visual impairment. |
| Amini et al., 2025.  Iran | To evaluate the impact of physical activity education, delivered via WhatsApp and based on the Health Belief Model (HBM), on the quality of life of perimenopausal women. | Study Type: Quasi-experimental.  Sample: 80 women aged 40–50 years.  Production: Development and distribution of educational packages via WhatsApp, including videos, 5 podcasts, posters, and specific training sessions.  Target Audience: Perimenopausal women.  Location: Isfahan, Iran Year: 2021 to 2022. | Physical activity;  Meaning of perimenopause and associated symptoms;  Consequences of physical inactivity;  Benefits of physical exercise during perimenopause;  Barriers and solutions for engaging in physical activity;  Self-efficacy techniques, exercise planning, and motivation. | The results indicated a significant increase in knowledge scores, Health Belief Model constructs, and physical activity levels in the intervention group, while no significant changes were observed in the control group.  Notably, menopause symptom scores decreased in the intervention group, particularly psychological symptoms, and there was a significant reduction in the overall quality of life score (P = 0.018). |
| Fallah-Karimi et al., 2025  Iran | To examine the effect of an online program like this on exclusive breastfeeding self-efficacy among primiparous mothers in Iran. | Study Type: Semi-experimental (non-randomized, with a control group).  Sample: 70 primiparous mothers.  Production: Online education delivered through a smartphone-accessible website containing short videos and podcasts. Educational content was published twice a week over eight sessions. The website included expert information and guidance on breastfeeding and proper breastfeeding techniques. 3 podcasts (30 min, 5 min, and 18 min).  Target Audience: Primiparous mothers during the postpartum period.  Location: Cities of Zahedan, Iran.  Year: 2024. | Breastfeeding  How to feed a baby with breast milk?  (1. When should you start breastfeeding?; 2. What should be the intervals between a baby’s feedings?; 3. How should you hold a baby during breastfeeding?; 4. How should the breast be positioned in the baby’s mouth?)  What are the common breast problems that breastfeeding mothers may face? (What are the most common nipple problems, breast engorgement, and mastitis?)  What factors are effective in ensuring the health of breastfeeding mothers? (1. What effect do a breastfeeding mother’s diet and rest have on her health?; 2. How can people around her and society support a breastfeeding mother?) | Online breastfeeding education significantly increased breastfeeding self-efficacy in the intervention group. |
| Hill et al., 2025,  Australia | To co-create resources aimed at reducing weight stigma in prenatal care and to evaluate healthcare professionals’ perspectives on these resources, considering their relevance to practice, strengths, and areas for improvement. | Study Type: Co-design project  Sample: Women with lived experience (n = 8), midwives (n = 16), and one obstetrician (n = 1)  Production: A short podcast developed to raise awareness about weight stigma in maternity care. The podcast included an interview between a midwife and a researcher specializing in weight stigma.  Target Audience: Healthcare professionals  Location: Monash University  Year: January 2023 to May 2024 | Weight stigma in prenatal care  Raising awareness about weight stigma in maternity care  (What weight stigma is; its prevalence; the consequences of weight stigma; examples of weight stigma in maternity care; and ways healthcare professionals can reduce weight stigma in maternity care settings.) | Production of a set of evidence-based resources considered valuable and relevant to practice by healthcare professionals. The resources included a video for the general public, created to foster empathy regarding lived experiences of weight stigma in maternity care; images representing women with different body types for use in clinic waiting rooms; a brief podcast to increase awareness of weight stigma in maternity care; and signs for the prenatal clinic encouraging healthcare professionals to consider weight stigma in all clinical interactions. |
| Christiansen et al., 2025  Denmark | To evaluate mothers’ use of the Healthy Together behavioral intervention, which aims to support new mothers in adopting a healthier lifestyle. Additionally, it assessed mothers’ experiences and attitudes when using the intervention. | Study Type: Mixed-methods  Sample: 34 women between 3 weeks and 6 months postpartum  Production: 6 podcasts. The intervention was co-created with healthcare professionals, mothers (intended users), and IT specialists to design an intervention that met women’s real needs.  Target Audience: Postpartum mothers  Location: Region of Southern Denmark  Year: June to December 2020 | Healthy lifestyle (diet and exercise)  Postpartum: Guiding mothers on how to maintain a healthy lifestyle after childbirth;  Diet: Information related to healthy eating;  Exercise: Information related to physical activity. | The aim of the module is to support new mothers in adopting a healthy lifestyle.  Push notifications and podcasts were used by 65% (n = 34) of participants and were therefore the most utilized content.  Podcasts were generally the preferred information channel.  The Healthy Together program has the potential to be a feasible tool to help women improve their lifestyle in the postpartum period. |
| Ignácio et al., 2025  Brazil | It presents Menopausando, a predominantly Portuguese-language digital platform designed to support women during the menopausal transition and postmenopause. | Study Type: Cross-sectional  Sample: Not addressed Production: 21 episodes ranging from 8 to 32 minutes  Target Audience: Women during the menopausal transition and postmenopause  Location: Gynecology Department, Universidade de São Paulo  Year: 2019 | Menopause  Hormone Replacement Therapy  Breast cancer  Cervical cancer  Sexual health  Endometriosis  Physical activity  Nutrition | Among the respondents, 83.3% were White, with a mean age of 54.3 years, and 78.8% expressed interest in a digital platform about menopause.  The platform reached 21,617 users on the website, 3,048 on Instagram, 133 on Facebook, and 1,126 on Spotify.  Facebook users were older, with a mean age of 51.3 years, compared to Spotify users, whose mean age was 45.6 years.  The highest number of Brazilian users was on the website, with 21,000 (97.1%) (across all groups), followed by users from other countries on the website and Instagram.  Menopausando effectively supports women during the menopausal transition and postmenopause through multiple digital channels. The results reveal trends and inequalities in digital access. |
| Vesco et al., 2024  USA | To use a flipped classroom model to deliver an innovative menopause curriculum for medical residents. | Study Type: Quasi-experimental  Sample: 200 residents (43 in obstetrics and gynecology, 86 in internal medicine, and 71 in family medicine)  Production: 6 podcast episodes  Target Audience: Residents in obstetrics and gynecology, internal medicine, and family medicine  Location: United States  Year: 2019 and 2020 | Menopause | Residents who listened to all six podcast episodes in full and those who attended the in-person session demonstrated greater gains.  Residents rated the podcast format as convenient and effective compared with an equivalent amount of reading.  The combination of a podcast with a classroom discussion proved effective in improving knowledge about menopause. |
| Ogunmodede et al., 2024  Nigeria | To compare two educational tools for teaching breast self-examination (BSE) to adolescents. | Study type: quasi-experimental  Sample: 276 participants; 161 received instruction through the traditional method (TM), that is, via a lecture-based class, and 115 received instruction through the modern method (MM), by viewing a podcast with lessons on breast self-examination (BSE).  Production: Not applicable.  Target Audience: Adolescents  Location: Public secondary schools in two distinct zones (the cities of Ogbomoso and Oyo) in Oyo State, southwestern Nigeria.  Year: December 2022 and April 2023. | BSE | The mean gains in knowledge and practice of breast self-examination (BSE) before and after the intervention improved when using the modern method (podcast).  The modern method (podcast) demonstrated superior effectiveness in teaching BSE knowledge and practice among adolescents compared with the traditional method. |
| Khairat, 2024  Egypt | To evaluate the effectiveness of medical podcasts as a new learning tool for developing awareness of Egyptian women’s health. | Study Type: Educational intervention  Sample: 50 postgraduate students  Production: *“Vaginal Infections”* is a medical podcast on vulvovaginal diseases created by the authors of this study.  Target Audience: Students, physicians, and patients  Location: Faculty of Arts, Suez Canal University  Year: 2024 | Vaginal infections | The podcast increased knowledge about gynecological diseases and proved to be an effective learning tool for healthcare students with a focus on women’s health.  This study emphasizes the role of podcasts as a valuable educational resource for society. The success of initiatives like this is expected to strengthen efforts to address the lack of knowledge among healthcare professionals regarding various diseases. |
| Mangum; Agarwal, 2024  USA | A reproductive endocrinologist, Dr. Sanjay K. Agarwal, and a patient advocate, Tara Mangum, share their perspectives on the diagnosis and treatment of endometriosis. They also discuss combination therapy with relugolix as a treatment option for patients with endometriosis. | Study Type: Qualitative (interview)  Sample: Not applicable (interview with a specialist and patient report)  Production: Podcast episode featuring discussions and interviews  Target Audience: Women and healthcare professionals  Location: United States  Year: 2024 | Diagnosis and treatment of endometriosis | A podcast featuring patient and physician perspectives on the treatment of endometriosis and combination therapy with relugolix. |
| França; Correia, 2024  Brazil | To analyze the potential of using a podcast to promote self-care in patients with resistant hypertension. | Study Type: Cross-sectional  Sample: 40 patients with resistant hypertension; 35 (87.5%) female  Production: Informative podcast produced by a nursing student, with a duration of 2 minutes and 44 seconds  Target Audience: Patients with resistant hypertension  Location: Resistant Hypertension Outpatient Clinic, Hospital Universitário Antônio Pedro, Universidade Federal Fluminense  Year: January to February 2022 | Self-care in Systemic Arterial Hypertension | The podcast was considered a promising strategy, as it promotes reflection, interaction, and greater patient autonomy in self-care.  Participants demonstrated increased engagement and awareness of the importance of self-care for hypertension management.  The podcast has potential as a strategy to promote self-care among patients with resistant hypertension. |
| Ó Caoilte et al., 2024  Ireland | To understand what listeners of mental health podcasts gain from this medium. | Study Type: Mixed-methods  Sample: Men (n = 207), women (n = 498), non-binary individuals (n = 14), and 3 participants who chose not to disclose their gender  Production: Not addressed  Target Audience: Men and women  Location: Ireland  Year: Not addressed | Mental health | The thematic analysis identified five main themes: accessibility, mental health knowledge, potential challenges, support, and lived experiences.  The accessibility of the material and discussions with professionals and individuals with lived experience were highlighted as key points.  The results indicate that podcasts influence the development of mental health literacy, reduce stigma, and increase help-seeking. Given the challenges in accessing services in underserved populations, there is potential for the use of podcasts in rural areas. |
| Fine et al., 2023  USA | To analyze the availability, content, and authorship of podcasts on fertility and to compare those presented by physicians with those presented by patients. | Study Type: Evaluative  Sample: 133 podcasts related to the term “fertility” across 10 podcast platforms (Anchor, Apple Podcasts, Spotify, Google Podcasts, Podbean, Overcast, Stitcher, Breaker, Castbox, and RadioPublic)  Production: Not applicable  Target Audience: Patients, holistic healthcare professionals, and physicians  Location: Stony Brook, New York  Year: 2020 | Fertility  Patient education  Fertility knowledge  Assisted reproduction with egg donation | When comparing podcasts presented by physicians with those presented by patients, there was no difference in overall content, episode frequency, number of episodes, or listener ratings.  There are many podcasts on fertility, most of which are presented by patients sharing their personal experiences on the topic.  Understanding the landscape of fertility podcasts will allow physicians to identify areas of interest and create content that educates and strengthens connections with patients. |
| Cai et al., 2023  USA | To evaluate the effect of an educational podcast on patient satisfaction and sense of control during childbirth. | Study type: RCT  Sample: Low-risk nulliparous women with singleton pregnancies. 153 participants.  Control group (CG) prenatal education: 75  Intervention group (IG) podcast education: 78.  Production: 7 podcasts. Three obstetricians, one midwife, one anesthesiologist, and two maternal-fetal medicine specialists. Duration: 10 to 20 minutes.  Target audience: Pregnant women  Location: Philadelphia, Pennsylvania  Year: July 2021 to January 2022 | Anesthesia for labor;  Complications of labor and delivery;  Normal labor;  Reasons for performing a cesarean section;  Second stage of labor;  Postpartum recovery;  Induction of labor. | Patients in the podcast group had higher median scores for satisfaction with childbirth compared to the control group.  When restricting the analysis to patients who underwent labor induction, the scores on the Labor Autonomy Scale were significantly higher in the podcast group, representing a greater perception of control.  The satisfaction score with childbirth was not different between the groups.  The most downloaded podcasts were about induction and anesthesia during labor. More than 95% of participants would recommend the podcasts to family and friends. |
| Ashai et al., 2023  USA | To evaluate the quality, comprehensibility, practical applicability, accuracy, and commercial bias of podcasts about stress urinary incontinence (SUI). | Study type: Cross-sectional  Sample: 100 podcasts related to SUI (Stress Urinary Incontinence) on the Stitcher platform. Average duration: 49 minutes  Production: Not applicable  Target audience: Physicians and general public  Location: USA  Year: May to August 2020 | SUI  Pelvic floor muscle training (PFMT)  Surgery for SUI | The treatments discussed included TMAP and surgery.  Most podcasts related to SUI are of low quality.  There is incorrect information in these podcasts related to treatment.  Podcasts that addressed surgery were more likely to discuss shared decision-making.  Podcasts with incorrect information had the highest average ratings. |
| Keenga et al., 2023  USA |  | Study type: qualitative  Sample: 17 listeners of The Sex Wrap, aged between 20 and 25 years.  Production: The Sex Wrap podcast is released in weekly episodes of approximately 20 to 45 minutes, offering listeners diverse and evidence-based information on sexual health.  Target audience: teenagers  Location: USA | Sex education | Podcast-based interventions, such as The Sex Wrap, can play a key role in the sexual education process for young people and should be considered for incorporation into other forms of sexual education. |
| Venable; Al-Hendy 2023.  USA | In this podcast, a Professor of Obstetrics and Gynecology from the University of Chicago (Dr. Ayman Al-Hendy) and the CEO of the Fibroid Foundation (Sateria Venable) share their perspectives on the diagnosis and treatment of uterine fibroids (UFs), including common treatments (such as myomectomy or hysterectomy). They also discuss combination therapy with Relugolix for UFs, indicating who may benefit from this treatment and how it works. | Study type: (interview with an expert)  Sample: not applicable  Production: a podcast on advancements in therapy, UF: a podcast about patient and physician perspectives on clinical treatment and a new drug therapy (combination therapy with relugolix).  Target audience: Healthcare professionals, patients with uterine fibroids  Location: USA  Year: 2023 | Diagnosis and treatment of UF (myomectomy, hysterectomy, and combination therapy with Relugolix) | This podcast is interpreted as scientific content, exploring the opinions and clinical insights of its participants. |
| Brufsky; Gallagher, 2023.  USA | To discuss how real-world data from heterogeneous patient populations can complement clinical trial data, aiding in therapeutic decision-making for patients with metastatic breast cancer that is hormone receptor-positive and human epidermal growth factor receptor 2-negative (HR+/HER2-). | Study type: Podcast commentary/transcription.  Sample: Initial: 2,888 patients (from a database of over 3 million).  After statistical adjustment: 2,709 patients, with 1,572 in the palbociclib + AI group and 1,137 in the AI-only group.  Production: A podcast offering a comprehensive overview of the P-REALITY X study, including its design, key findings and clinical implications, and how this information can be integrated into patient conversations.  Target audience: Healthcare professionals, especially oncologists, physicians, and researchers interested in metastatic breast cancer treatments and real-world evidence.  Location: USA  Year: 2023 | Treatment of metastatic breast cancer | The value of real-world studies in guiding treatment decisions for patients with metastatic breast cancer.  The study showed that HR+/HER2− metastatic breast cancer patients treated with palbociclib + aromatase inhibitor had greater overall survival (median of 49.1 months) compared to treatment with aromatase inhibitor alone (43.2 months).  There was a prolongation of time to disease progression (median of 19.3 vs. 13.9 months).  The benefits were observed in several subgroups, including older patients and those of different racial backgrounds. |
| Galeotti et al., 2023  United Kingdom | To determine the effectiveness of a brief, self-directed educational intervention in increasing nursing students' reported knowledge, skills, awareness, and confidence regarding pregnancy loss. | Study type: Cross-sectional  Sample: 244 first-year nursing students  Production: 82-minute podcast. The podcast content was developed and presented by a midwife, a nurse, and a psychologist. The podcast included three lived experiences (miscarriage, stillbirth, and termination of pregnancy due to fetal anomaly).  Target audience: First-year undergraduate nursing students (no significant prior experience in pregnancy loss care)  Location: Queen’s University Belfast, Northern Ireland, United Kingdom.  Year: 2022 | Pregnancy loss (miscarriage, stillbirth, and termination of pregnancy due to fetal anomaly)  Focus on hospital care and how it impacted your pregnancy loss journey, the grieving process, and recovery. | The educational intervention increased nursing students' perception of their knowledge, confidence, and skills in caring for families experiencing pregnancy loss. |
| Stovern K, 2023  USA | To analyze podcasts in order to shed light on issues affecting the well-being of women with gender disparities in access to healthcare. | Study type: Cross-sectional  Sample: 154 women aged 18 and older  Production: 2 episodes of less than 44 minutes each. The podcasts were presented in an interview format about the increasing rate of maternal morbidity and mortality in Missouri and across the country (The developed podcast is called "Why Am I Just Finding This Out?")  Target audience: Women from Missouri aged 18 and older, including healthcare professionals from any geographic location in the United States.  Location: Missouri  Year: 2023. | The problem of maternal mortality rates  Collaborative care to improve women's health | Podcasts have proven to be a viable tool for increasing knowledge in women's health, with high public acceptance.  More than 94% of participants indicated that listening to podcasts recommended by health professionals would help with learning.  The most searched topics include nutrition, exercise, menstrual cycles, menopause, and women's research.  Populations in different age groups show a preference for content in varied formats, with podcasts being most accessed by women aged 40 or older.  Podcasts can help reduce disparities by being accessible, convenient, and easy to understand, helping to promote autonomous behavioral change. |
| Souza, 2022.  Brazil | Develop an educational podcast to provide discharge instructions to patients in the postoperative period following a mastectomy. | Study type: Methodological  Sample: Women discharged from hospital after mastectomy.  Production: Educational podcast produced by a nurse, structured in three episodes, edited and published via digital media.  Target audience: Women discharged from hospital after mastectomy surgery.  Location: University hospital and digital platforms (social media, eduCAPES portal, QR Code).  Year: 2022 | Definition of breast cancer;  Mastectomy as part of breast cancer treatment;  Nursing guidelines for patients discharged from the hospital after mastectomy and for patients using a hemovac drain. | The podcast was structured to provide information about breast cancer, surgery, and nursing care upon hospital discharge, facilitating patient understanding and self-care.  Patients reported benefits in understanding care and greater confidence during self-management of care at home.  The podcast, as a teaching and learning tool combined with nursing care, allows for the distribution of correct guidance on a given care recommendation. This tool optimizes the transmission of knowledge in a simple and objective way, with the aim of safely encouraging patients to maintain their self-care at home with peace of mind, clarity, confidence, and security. |
| Wilcox et al., 2022.  USA | To describe the assessment methods and outcomes of a behavioral lifestyle intervention for overweight and obese African American and white women, spanning from pregnancy (≤ 16 weeks) to 6 months postpartum. | Study type: RCT  Sample: GI: (in-depth counseling session, phone calls, podcasts)  Production: 20 podcasts.  Target audience: Overweight or obese pregnant women of diverse races during pregnancy.  Location: United States  Year: 2022 | Behavioral strategies for habit change;  Physical activity during pregnancy;  Healthy diet and nutrition;  General recommendations during pregnancy;  Social support techniques and assistance. | The dose received and participants' evaluations of the in-depth counseling session and phone calls were more favorable than those of the podcasts.  More calls made and more podcasts downloaded were associated with less gestational weight gain.  The intervention significantly improved vegetable consumption and the percentage of whole grains. There was a decline in total physical activity, daily steps, and physical quality of life in both groups;  Improvements in diet quality were observed only in the diet group, with modest gains in physical activity and mental quality of life, the latter decreasing more in the intervention group. |
| Shaw et al., 2022  USA | To explore women's perceptions and engagement with the podcast 'Menopause: No Silencing', specifically exploring the mechanisms by which women connected with the health information presented through audio stories. | Study type: Qualitative  Sample: A diverse sample of 30 women aged 40 to 60 years  Production: Five 20-minute podcast episodes about how women coped with their menopause experiences, narrated by the women themselves with commentary from a healthcare professional. An additional sixth episode summarized the main points of the previous episodes.  Target audience: Women in menopause  Location: USA  Year: October 2020 to September 2022 | Menopause | This study suggests that a series of podcasts used to communicate health information about menopause was received positively.  Story-based podcasts are an engaging and effective way to learn about menopause.  The content is considered authentic, credible, and can influence health-related behaviors.  Podcasts should seek to increase engagement through the representation of diverse audiences. |
| Dmytryshyn; Selk, 2022  Canada and USA | To evaluate the effectiveness of the podcast "The Vulva Diaries" as a new learning tool for education on vulvovaginal diseases. | Study type: Educational intervention  Sample: 101 medical students and 46 residents (gynecology, family medicine, and neurology)  Production: "The Vulva Diaries" series: 39 episodes were published.  Target audience: General public. Although the series is publicly accessible, the episodes are primarily aimed at medical students and healthcare professionals involved in women's health.  Location: Canada and USA  Year: December 5, 2020 to June 5, 2021. | Genital herpes;  Lichen sclerosus  Prevention, diagnosis, treatment, and impact of various lower genital tract conditions related and unrelated to human papillomavirus (HPV), as well as dermatological disorders. | The podcast "The Vulva Diaries" increases knowledge about vulvovaginal diseases and is an effective learning tool for healthcare professionals in training in the field of women's health.  Medical students and residents rated their average preference for using podcasts compared to other resources.  This study emphasizes the role of podcasts as a valuable educational resource in gynecology. |
| Daniel, 2022,  Brazil | To develop and evaluate a podcast proposal as a tool for disseminating scientific information about motherhood and mothering, promoting dialogue between science and the lived experiences of mothers. | Study type: Methodological  Sample: Not applicable  Production: Pilot episode. 60 to 75 minutes. Featuring mothers and healthcare professionals  Target audience: Women who are mothers aged 25-55  Location: Uberlândia, Brazil  Year: 2022 | Breastfeeding  Importance of breastfeeding  Benefits of breastfeeding  Challenges of breastfeeding  Breastfeeding consulting  Human Milk Banks in Brazil  Maternal guilt | The episode intertwines real stories of mothers who experienced breastfeeding with scientific practices and data presented by experts.  The podcast enabled a dialogical exchange between mothers and science, promoting greater access to scientific information and personal experiences.  The production proved to be an effective tool for disseminating complex topics in an accessible and participatory way.  The importance of scientific knowledge in validating maternal practices and deconstructing ideal discourses of motherhood was highlighted.  Challenges such as mother engagement, production costs, and competition in the podcast market were identified. |
| Wachan, 2022  Brazil | To understand the process of subjectivity production among contemporary mothers and its relationship with a regime of truths called "real motherhood," based on statements conveyed in podcasts focused on the theme of motherhood. | Study type: Qualitative  Sample: 10 podcast channels related to the maternal niche, specifically the first episode published by each of them  Production: Spotify platform  Target audience: Women/mothers  Location: Uberlândia, Brazil  Year: 2022 | Motherhood  Challenges of motherhood  Postpartum period;  Maternal guilt;  Challenges of breastfeeding;  Motherhood and work;  Maternal fatigue and exhaustion  Motherhood during quarantine/pandemic  Resignation during pregnancy | Podcasts function as central spaces for the production of maternal subjectivity.  Although the discourse is about "real motherhood," it ends up being directed primarily at a specific profile: cisgender, white, middle-class women. The study points out a lack of diversity in class, gender, and race in the discourses analyzed. |
| Saldanha, 2022  Brazil | To develop and validate podcast content about the physiological changes of pregnancy and the phases of embryonic and fetal human development. | Study type: Methodological  Sample: Not applicable  Production: Educational and informative podcasts, in audio format, with an average duration of 5 to 10 minutes per episode, planned for later recording and distribution in health services.  Target audience: Pregnant women  Location: Caicó, Rio Grande do Norte, Brazil.  Year: 2022 | Embryonic human development;  Fetal development;  Physiological and anatomical changes in the maternal organism | All podcast content items were validated with a Content Validity Index (CVI) > 0.80 after two rounds of evaluation.  The material was considered clear, relevant, and appropriate for the target audience.  Subsequently, the podcasts will be recorded and distributed to local health units. |
| Edwards et al., 2021  USA | To understand the impact of the podcast "Menopause: No Silence" on women's knowledge, understanding, and communication practices related to menopause. | Study type: qualitative  Sample: 30 women aged 40 to 60 years  Production:  Target audience: women in perimenopause and postmenopause  Location: USA  Year: 2021 | Menopause | "Reshaping Menopause" describes the impact of the podcast, in which women reflect on the value of communication among women, question and reassess the stigmatization of menopause, and discuss ways to promote positive behavioral changes in their lives.  The podcast "Menopause: No Silence" helped women learn about the experience of menopause, have a greater sense of belonging to a female community, and feel empowered to make changes in their own lives.  Sharing stories through podcasts has potential as an accessible and impactful means to educate women and reduce the widespread stigma associated with menopause. |
| Militello et al., 2021  USA | To assess the feasibility of using voice technology to support perinatal health and childcare practices. | Study type: Mixed methods  Sample: 19 pregnant women (between 17 and 36 weeks of gestation)  Production: Mini podcasts (lasting 3 to 8 minutes), totaling 239 episodes  Target audience: Women in the perinatal period  Location: Nationwide Children’s Hospital, Columbus, Ohio, USA  Year: 2021 | Pregnancy  Baby's sleep routines  Newborn care (Oral hygiene, vaccination, and pacifier)  Breastfeeding  Formula feeding  Parenting | The results demonstrate the short-term feasibility of disseminating evidence-based perinatal support through podcasts and collecting voice data from women in the perinatal period. |
| Reese et al., 2021  USA | To evaluate the feasibility, acceptability, and preliminary effects of a mobile learning (mLearning) intervention (Enhancing Sexual Health and Strengthening Relationships through Education [iSHARE]) designed to increase the knowledge, beliefs, and comfort of physicians treating breast cancer when discussing their patients' sexual health concerns. | Study type: Single-arm pilot trial  Sample: 30 healthcare professionals (clinical oncologists, advanced practice nurses, and physician assistants)  Production: Two-part educational podcast series, with each episode containing a 30-minute interview with guest experts  Target audience: Medical oncologists and nurses  Location: USA  Year: February to October 2020 | Sexual health  Breast cancer  Sexual problems related to breast cancer  Treatments/solutions for sexual problems in women with breast cancer | The results showed positive trends of improvement in physicians' knowledge, beliefs, and comfort when addressing sexual health issues.  Physicians reported important lessons learned, including adopting a proactive approach to discussing sexual health issues, normalizing the topic, addressing vaginal health, conveying the message that help is available, and assessing sexual health issues in patients from different backgrounds.  Breast cancer specialists were receptive to participating in the iSHARE intervention and found it useful. |
| Ferreira, 2021  Brazil | To assess the challenges faced by breastfeeding consultants and postpartum women who conducted online consultations during the Covid-19 pandemic, and to examine their experiences during this period. | Study type: descriptive  Sample: 52 breastfeeding consultants and 40 postpartum women participating in the research.  Production: The podcasts were developed as an educational product of the dissertation (available on Spotify).  Target audience: Pregnant women, postpartum women, healthcare professionals, and consultants.  Location: Porto Alegre, Brazil  Year: 2021 | Breastfeeding (breastfeeding during the Covid-19 pandemic) | Technical products were developed to guide breastfeeding during the Covid-19 pandemic (podcasts, educational booklet on the subject), in addition to providing support materials for the work of these lactation consultants (online questionnaires to guide breastfeeding consultations). |
| Cai et al., 2020  USA | We describe the development and implementation of a series of medical education podcasts produced by residents for the learning of obstetrics and gynecology (Ob-Gyn) residents, and demonstrate the feasibility, sustainability, and acceptance of this series.. | Study Type: Descriptive  Sample:  Production: 60 podcast episodes. Most episodes featured the participation of two third-year residents and a reviewing obstetrician-gynecologist, and some episodes also featured a specialist in another area. 10 to 30 minutes in length. The educational guidelines of the Council on Resident Education in Obstetrics and Gynecology (CREOG) were used to create a weekly study podcast for Obstetrics and Gynecology residents over 10 months.  Target Audience: Obstetrics and Gynecology residents.  Location: USA  Year: September 30, 2018 and July 28, 2019 | Obstetrics (Prenatal care; Obstetric complications; Care during childbirth; Newborn care; Postpartum care)  Gynecology (Urogenital Tract and Breast Disorders; First Trimester Pregnancy Failure; Intensive Care; Cervical Neoplasia; Female Malignant Tumors; Breastfeeding; Gynecological Disorders; Treatment of Non-Gynecological Conditions and Disorders; Perimenopause and Menopause)  Geriatric Care  Perioperative Care  Vaccines  Domestic Violence and Armed Violence  Maternal-Fetal Medicine  Oncology  Contraception  Pediatric and Adolescent Obstetrics and Gynecology  Reproductive Endocrinology and Infertility  Female Pelvic Medicine and Reconstructive Surgery | A weekly Gynecology and Obstetrics podcast created by residents, based on CREOG guidelines and with content review by faculty experts, proved viable over a 10-month period. The podcasts were highly accepted and downloaded nearly 175,000 times by people from 100 countries and all 50 states. |
| Dahl, 2018  USA | To test the effects of a mobile application and a study website for a gestational weight gain intervention targeting pregnant women living in the United States.. | Study type: RCT  Sample: 140 healthy pregnant women  Output: 10 informative podcasts and weekly health tips for pregnancy;  Target audience: pregnant women  Location: USA  Year: 2018 | Healthy Eating and Physical Activity  Healthy Gestational Weight Gain  Healthy eating during pregnancy;  Safe physical activity during pregnancy;  Behavioral strategies for habit change;  Stress Reduction and Management | Although the intervention's effect on reducing excessive gestational weight gain was not significant, the study demonstrated potential for facilitating healthy behavioral changes through group goal setting and self-monitoring using a mobile application. |
| Wilcox et al., 2018  USA | To examine the impact of a theory-based lifestyle intervention on gestational weight gain, postpartum weight loss, and related maternal and infant outcomes, as well as to examine racial differences in these outcomes. | Study Type: RCT  Sample: Overweight or obese African American and white women, up to 16 weeks pregnant. GI: Two in-depth counseling sessions (early pregnancy and postpartum), telephone counseling, behavioral podcasts, and social media support, focusing on weight self-monitoring and increasing physical activity and healthy eating practices, guided by Social Cognitive Theory. GC: Monthly correspondence and an equivalent number of podcasts on topics unrelated to weight.  Production: Participants receive 10 podcasts on behavior during pregnancy and 16 during the postpartum period.  Target Audience: Overweight and obese pregnant women.  Location: Obstetrics and gynecology clinics in South Carolina.  Year: 2018 | Recommended weight gain during pregnancy;  Healthy diet;  Physical activity;  Gradual weight loss. | Evaluation of the effectiveness of a behavioral lifestyle intervention program to reduce excessive weight gain during pregnancy and promote postpartum weight loss in overweight/obese women. |
| Legro, 2015.  USA | This paper discusses recommendations for the diagnosis and treatment of polycystic ovary syndrome (PCOS) based on clinical practice guidelines and addresses the challenges of diagnosing PCOS in specific age groups. | Study type: Qualitative (interview with an expert)  Sample: Not applicable  Production: Scientific podcast linked to the journal BMC Medicine, accompanied by an edited transcript and made available as supplementary material online.  Target audience: Healthcare professionals and students  Location: USA  Year: 2015 | Diagnosis and treatment of PCOS  Infertility  Hirsutism  Menstrual disorders | Strengthening the use of the Rotterdam criteria for the diagnosis of PCOS, excluding differential diagnoses.  Importance of individualizing treatment according to the patient's complaint.  Evidence that letrozole showed a higher live birth rate compared to clomiphene in women with PCOS and infertility.  Recognition of the limitations of lifestyle interventions and the use of metformin as first-line treatment for reproductive manifestations.  Indication of new biomarkers (such as anti-Müllerian hormone) and genetic advances as future perspectives. |
| Turner-McGrievy et al., 2009  USA | A weight loss podcast developed based on health behavior theories will produce greater weight loss than a currently available podcast that is not based on theories. | Study type: RCT  Sample: Overweight men and women (body mass index, 25–40 kg/m²) (n = 78)  Output: 24 episodes of a pre-existing weight loss podcast (GC: control podcast) or a weight loss podcast based on social cognitive theory (SCT) developed by the researchers (GI: enhanced podcast) over 12 weeks.  Target audience: Overweight women and men  Location: North Carolina, United States  Year: 2008 | Weight Loss | Participants in the GI (n = 41) showed greater weight and BMI reduction than the GC (n = 37), as well as greater knowledge, elaboration, and user control related to weight loss and lower cognitive load.  The use of theory-based behavioral podcasts can be an effective way to promote weight loss. |

**References**

1. Parnell J, Daly D, Duff J, Walsh A. Quality of health information for women with epilepsy from preconception to postpartum: An evaluation of online resources. Epilepsy Behav. 2025;172:110656. doi: 10.1016/j.yebeh.2025.110656
2. Brito LKT**.** Podcast educativo sobre amamentação para pessoas com deficiência visual: promoção da saúde e empoderamento [dissertação]. Redenção: Universidade da Integração Internacional da Lusofonia Afro-Brasileira; 2025. Disponível em: https://sucupira-legado.capes.gov.br/sucupira/public/consultas/coleta/trabalhoConclusao/viewTrabalhoConclusao.jsf?popup=true&id_trabalho=16809425
3. Amini M, Shekarchizadeh P, Pirzadeh A. Can mobile software-based physical activity education enhance the quality of life in perimenopausal women? J Educ Health Promot. 2025;14:85. doi: 10.4103/jehp.jehp_1919_23.
4. Fallah-Karimi S, Robabi H, Khalilzadeh-Farsangi Z. Impact of web-based breastfeeding education on breastfeeding self-efficacy and exclusive breastfeeding rates among primiparous mothers in iran: a quasi-experimental study. BMC Pregnancy Childbirth. 2025;25(1):797. doi: 10.1186/s12884-025-07955-9
5. Hill B, Hailu H, Jenkinson B, Rakic S, Nagpal TS, Boyle JA, Sheehan P, Darlison S, Skouteris H. Co-design and clinician evaluation of resources to address weight stigma in antenatal care. BMC Pregnancy Childbirth. 2025;25(1):263. doi: 10.1186/s12884-025-07327-3
6. Christiansen PK, Kjær T, Vinter CA, Rothmann MJ, Skjøth MM, Draborg E. Behavioral Change Intervention to Promote a Healthier Postpartum Lifestyle: Mixed Methods Pilot Study. JMIR Form Res. 2025;9:e69391. doi: 10.2196/69391
7. Ignácio MO, Martins MG, Portella CFS, Santiago AE, Zangirolami-Raimundo J, Baracat EC, Soares JM Jr, Sorpreso ICE. “Menopausando”: a digital health education platform for women in the transition to menopause and postmenopause. Climacteric. 2025;1-7. doi:10.1080/13697137.2025.2548798
8. Vesco KK, Brooks NB, Francisco MC, Leo MC, Anderson TM, Kalter M, Clark AL. Resident training to optimize patient-focused menopause management: a multispecialty menopause curriculum to enhance knowledge and preparedness. Menopause. 2024;31(2):93-100. doi:10.1097/GME.0000000000002291
9. Ogunmodede EO, Ajala DE, Aluko JO, et al. Promoting breast health among female adolescents: a comparative analysis of the effects of two didactic tools on knowledge and practice regarding breast self-examination in Southwest Nigeria. BMC Cancer. 2024;24(1):1183. doi:10.1186/s12885-024-12949-5
10. Khairat P. The Effectiveness of Medical Podcasts in Raising Women’s Health Awareness. Clinical Psychology and Special Education. 2024;13(1):117–127. https://doi.org/10.17759/cpse.2024130108
11. Mangum T, Agarwal SK. A Podcast on Patient and Physician Perspectives on the Management of Endometriosis and Relugolix Combination Therapy. Adv Ther. 2024;41(12):4369-4376. doi: 10.1007/s12325-024-02970-2
12. França BC, Correia DMS. Podcast: uma estratégia para promoção do autocuidado do hipertenso resistente. Rev. enferm. UFPE on line. 2024;18:e263070. doi:doi:10.5205/1981-8963.2024.263070
13. Ó Caoilte N, Lambert S, Murphy R. 'There's no waiting list, just press play': listeners' experiences of mental-health-related podcasts. Rural Remote Health. 2024;24(2):8213. doi: 10.22605/RRH8213
14. Fine E, Peyser A, Abittan B, Mullin C, Goldman RH. The voice of infertility: a review of fertility podcasts. Hum Fertil (Camb). 2023;26(2):284-288. doi: 10.1080/14647273.2022.2082328
15. Cai F, McCabe M, Srinivas SK. A randomized trial assessing the impact of educational podcasts on personal control and satisfaction during childbirth. Am J Obstet Gynecol. 2023;228(5):592.e1-592.e10. doi:10.1016/j.ajog.2023.01.021
16. Ashai S, Pace L, Malik RD. Assessment of Stress Urinary Incontinence-Related Podcasts on Stitcher. Urology. 2023;174:79-85. doi: 10.1016/j.urology.2023.01.044
17. Keegan J, Cooper SC, Porter A, Ciervo C, Khalid R. Unlearning and relearning sexuality: a qualitative exploration of The Sex Wrap, a sex education podcast. Sex Health. 2023;20(6):531-537. doi: 10.1071/SH23109
18. Venable SR, Al-Hendy A. Uterine fibroids: a podcast on patient and physician perspectives on medical management and a new medical therapy (relugolix combination therapy). Advances in Therapy**.** 2023;40(10):4127-4133. doi:10.1007/s12325-023-02595-x
19. Brufsky A, Gallagher C. P-REALITY X: A Real-World Analysis of Palbociclib Plus an Aromatase Inhibitor in HR+/HER2- Metastatic Breast Cancer-A Podcast. Target Oncol. 2023;18(3):321-326. doi: 10.1007/s11523-023-00968-4
20. Galeotti M, Heaney S, Robinson M, Aventin Á. Evaluation of a pregnancy loss education intervention for undergraduate nursing students in Northern Ireland: A pre- and post-test study. BMC Nurs. 2023;22(1):268. doi: 10.1186/s12912-023-01408-4
21. Stovern K. "Feasibility of using podcasts for knowledge dissemination to reduce health disparities for women targeting preventative initiatives and lifestyle medicine interventions" (2023). Doctor of Nursing Practice Scholarly Project. 106. [acesso 06 de janeiro de 2026]. Available from: https://digitalcommons.pittstate.edu/dnp/106
22. Souza MF. Podcast como ferramenta de orientação às pacientes em pós-operatório de mastectomia: estudo metodológico [dissertação]. Niterói: Universidade Federal Fluminense; 2022.
23. Wilcox S, Dahl AA, Boutté AK, Liu J, Day K, Turner-McGrievy G, Wingard E. Process evaluation methods and results from the Health in Pregnancy and Postpartum (HIPP) randomized controlled trial. BMC Pregnancy Childbirth. 2022;22(1):794. doi: 10.1186/s12884-022-05107-x
24. Shaw PA, Sumner AL, Halton CC, Bailey SC, Wolf MS, Andrews EN, Cartwright T. "You're more engaged when you're listening to somebody tell their story": A qualitative exploration into the mechanisms of the podcast 'menopause: unmuted' for communicating health information. Patient Educ Couns. 2022;105(12):3494-3500. doi: 10.1016/j.pec.2022.09.003
25. Dmytryshyn J, Selk A. Learning on the Go: Assessing Knowledge Gained From Medical Podcasts Created for Vulvovaginal Disease Education. J Low Genit Tract Dis. 2022;26(2):164-168. doi: 10.1097/LGT.0000000000000654
26. Daniel RG. A mãe no meio: proposta de podcast de divulgação científica sobre maternidade e maternagem. 2022. 90 f. Dissertação (Mestrado Profissional em Tecnologias, Comunicação e Educação) - Universidade Federal de Uberlândia, Uberlândia, 2022. doi: <http://doi.org/10.14393/ufu.di.2022.273>
27. Walchan I. Cuidar de si, cuidar do outro: a subjetivação das mulheres contemporâneas na busca de uma “maternidade real” em podcasts. 2022. 145 f. Dissertação (Mestrado em Estudos Linguísticos) - Universidade Federal de Uberlândia, Uberlândia, 2022. doi: <http://doi.org/10.14393/ufu.di.2021.525>
28. Saldanha FHP. Podcasts sobre desenvolvimento humano embrionário e fetal e modificações fisiológicas e anatômicas no organismo materno: validação de conteúdo. Universidade Federal do Rio Grande do Norte. 2022. Available from: <https://repositorio.ufrn.br/handle/123456789/49536>
29. Edwards AL, Shaw PA, Halton CC, Bailey SC, Wolf MS, Andrews EN, Cartwright T. “It just makes me feel a little less alone”: a qualitative exploration of the podcast *Menopause: Unmuted* on women's perceptions of menopause. Menopause. 2021;28(12):1374-1384. doi:10.1097/GME.0000000000001855
30. Militello L, Sezgin E, Huang Y, Lin S. Delivering Perinatal Health Information via a Voice Interactive App (SMILE): Mixed Methods Feasibility Study. JMIR Form Res. 2021;5(3):e18240. doi: 10.2196/18240
31. Reese JB, Zimmaro LA, Bober SL, Sorice K, Handorf E, Wittenberg E, El-Jawahri A, Beach MC, Wolff AC, Daly MB, Izquierdo B, Lepore SJ. Mobile Technology-Based (mLearning) Intervention to Enhance Breast Cancer Clinicians' Communication About Sexual Health: A Pilot Trial. J Natl Compr Canc Netw. 2021;19(10):1133-1140. doi: 10.6004/jnccn.2021.7032
32. Ferreira CA. Consultoria de aleitamento materno on-line: proposta ao enfrentamento da pandemia da COVID-19. 2021.Dissertação. Universidade Federal do Rio Grande do Sul. Disponível em: https://lume.ufrgs.br/handle/10183/236407
33. Cai F, Burns RN, Kelly B, Hampton BS. CREOGs Over Coffee: Feasibility of an Ob-Gyn Medical Education Podcast by Residents. J Grad Med Educ. 2020;12(3):340-343. doi: 10.4300/JGME-D-19-00644.1
34. Dahl AA. (2018). Healthy Motivations for Moms-To-Be (Healthy MoM2B) Study: A Mobile Health Intervention Targeting Gestational Weight Gain among U.S. Women. (Doctoral dissertation). Retrieved from https://scholarcommons.sc.edu/etd/4862
35. Wilcox S, Liu J, Addy CL, Turner-McGrievy G, Burgis JT, Wingard E, Dahl AA, Whitaker KM, Schneider L, Boutté AK. A randomized controlled trial to prevent excessive gestational weight gain and promote postpartum weight loss in overweight and obese women: Health In Pregnancy and Postpartum (HIPP). Contemp Clin Trials. 2018;66:51-63. doi:10.1016/j.cct.2018.01.008.
36. Legro R. Diagnosis and treatment of polycystic ovary syndrome (PCOS): an interview with Richard Legro. BMC Med. 2015;13:64. doi: 10.1186/s12916-015-0299-2
37. Turner-McGrievy GM, Campbell MK, Tate DF, Truesdale KP, Bowling JM, Crosby L. Pounds Off Digitally study: a randomized podcasting weight-loss intervention. Am J Prev Med. 2009;37(4):263-9. doi: 10.1016/j.amepre.2009.06.010
